# Smooth Muscle-Like Cells Generated from Human Mesenchymal Stromal Cells Display Marker Gene Expression and Electrophysiological Competence Comparable to Bladder Smooth Muscle Cells

**DOI:** 10.1371/journal.pone.0145153

**Published:** 2015-12-16

**Authors:** Juliane Brun, Katrin A. Lutz, Katharina M. H. Neumayer, Gerd Klein, Tanja Seeger, Tatiana Uynuk-Ool, Katharina Wörgötter, Sandra Schmid, Udo Kraushaar, Elke Guenther, Bernd Rolauffs, Wilhelm K. Aicher, Melanie L. Hart

**Affiliations:** 1 Clinical Research Group KFO 273, Department of Urology, University of Tübingen, Tübingen, Germany; 2 Center for Medical Research, University Medical Clinic, Department II, University of Tübingen, Tübingen, Germany; 3 Siegfried Weller Institute for Trauma Research, Laboratory for Molecular Biomechanics, University of Tübingen, Tübingen, Germany; 4 NMI Natural and Medical Sciences Institute at the University of Tübingen, Department of Electrophysiology, Reutlingen, Germany; University of Nevada School of Medicine, UNITED STATES

## Abstract

The use of mesenchymal stromal cells (MSCs) differentiated toward a smooth muscle cell (SMC) phenotype may provide an alternative for investigators interested in regenerating urinary tract organs such as the bladder where autologous smooth muscle cells cannot be used or are unavailable. In this study we measured the effects of good manufacturing practice (GMP)-compliant expansion followed by myogenic differentiation of human MSCs on the expression of a range of contractile (from early to late) myogenic markers in relation to the electrophysiological parameters to assess the functional role of the differentiated MSCs and found that differentiation of MSCs associated with electrophysiological competence comparable to bladder SMCs. Within 1–2 weeks of myogenic differentiation, differentiating MSCs significantly expressed alpha smooth muscle actin (αSMA; *ACTA2*), transgelin (*TAGLN*), calponin (*CNN1*), and smooth muscle myosin heavy chain (SM-MHC; *MYH11*) according to qRT-PCR and/or immunofluorescence and Western blot. Voltage-gated Na^+^ current levels also increased within the same time period following myogenic differentiation. In contrast to undifferentiated MSCs, differentiated MSCs and bladder SMCs exhibited elevated cytosolic Ca^2+^ transients in response to K^+^-induced depolarization and contracted in response to K^+^ indicating functional maturation of differentiated MSCs. Depolarization was suppressed by Cd^2+^, an inhibitor of voltage-gated Ca^2+^-channels. The expression of Na^+^-channels was pharmacologically identified as the Na_v_1.4 subtype, while the K^+^ and Ca^2+^ ion channels were identified by gene expression of *KCNMA1*, *CACNA1C* and *CACNA1H* which encode for the large conductance Ca^2+^-activated K^+^ channel BK_Ca_ channels, Ca_v_1.2 L-type Ca^2+^ channels and Ca_v_3.2 T-type Ca^2+^ channels, respectively. This protocol may be used to differentiate adult MSCs into smooth muscle-like cells with an intermediate-to-late SMC contractile phenotype exhibiting voltage-gated ion channel activity comparable to bladder SMCs which may be important for urological regenerative medicine applications.

## Introduction

The smooth muscle of the urinary tract is subjected to a variety of diseases and pathologies. Injury, loss or degeneration of urinary tract smooth muscle can severely alter micturition patterns and hence the quality of life. Although a number of treatments are available, limitations such as complications or recurrence have motivated investigators to seek out therapies that may provide safer and longer-lasting outcomes by repairing the damaged tissue itself.

In cell-based tissue engineering of the human bladder autologous bladder cells are sometimes used as a cell source. While clinical trials using autologous bladder smooth muscle cells (SMCs) show that such cell-based approaches seem promising for bladder tissue regeneration [1–3], one problem with this approach is that bladder cells are unavailable in some patients (e.g. diseased or malignant bladders). In such cases bladder cells cannot be used for regenerative therapies. Human mesenchymal stromal cells (MSCs) are multipotent adult progenitor cells capable of differentiating into a variety of cell types and have regenerative potential [4,5]. Over the past decade injection of MSCs has been assessed in humans for the treatment of urinary incontinence [6–9]. Although these highly valuable studies have shown an improvement in urethral closure pressure of the urethral sphincter, there is no clear evidence that these cells reconstruct the muscle. Moreover, some studies investigating stem cell therapy for urinary incontinence or bladder dysfunction have shown that only a small fraction of the undifferentiated MSCs have differentiated into smooth muscle *in situ* [10–12]. The use of MSCs differentiated toward a SMC phenotype may provide an alternative for investigators interested in regenerating urinary tract organs.

The contractile property of smooth muscle plays an important functional role in urinary tract organs by allowing dynamic changes in luminal volume such as in the urinary bladder where smooth muscle in the wall relaxes during filling and contracts forcefully to expel urine during micturition [13] or in supporting urethral storage pressure during bladder filling and storage which is partially aided by the internal urethral sphincter smooth muscle [14]. Ion channels in the smooth muscle membranes are essential in determining these functional properties [13].

There are a number of methodologies for differentiating MSCs to SMCs *in vitro*. For example, co-culture of MSCs with bladder SMCs or conditioned medium from those cells have been used to differentiate MSCs into SMCs [15–17]. The experimental differentiation of MSCs toward the smooth muscle lineage has also been described using various combinations of biochemical factors such as transforming growth factor beta 1 (TGF-β1) [17–25], L-ascorbic acid (also known as vitamin C) [21,22,25,26] or platelet derived growth factor (PDGF-BB) [17,22,23,26]. Moreover various human sources including MSCs isolated from bone marrow, adipose tissue and hair follicles were differentiated *in vitro* to generate smooth muscle-cell like cells. However, the electrophysiological competence of differentiated cells was not investigated in those studies. Therefore the aim of this study was to measure the effects of good manufacturing practice (GMP)-compliant expansion followed by myogenic differentiation of human MSCs using TGF-β1, ascorbic acid and PDGF-AB on the expression of a range of contractile myogenic markers in addition to the electrophysiological properties to assess the functional role of differentiated MSCs in comparison to bladder smooth muscle cells.

## Materials and Methods

Human tissue used in this study was obtained according to institutional approval from the University of Tübingen research ethics committee (623/2013BO2) and with written informed donor consent obtained for the use of this sample in research.

### Isolation and Expansion of Human MSCs

Bone marrow was obtained from the proximal femur during routine hip replacement by the BG Trauma Clinic (Tübingen, Germany). Bone marrow was washed with PBS, centrifuged at 150 x g (10 min at RT), the supernatant was discarded and cells were resuspended in PBS. MSCs were isolated using a Ficoll^®^ density gradient fractionation (density 1.077 g/mL, GE Healthcare Life Sciences, Uppsala, Sweden; 400 x g, 30 min, RT). The mononuclear cell layer was harvested, washed with PBS and seeded in T75 flasks (BD Falcon, Heidelberg) and expanded in GMP-compliant expansion medium: DMEM low glucose (Sigma-Aldrich, Taufkirchen, Germany), 1000 IU heparin (Carl Roth, Karlsruhe, Germany), 25 mM HEPES (Lonza, Basel, Switzerland), 5% human plasma (TCS Biosciences, Buckingham, UK), 5% human pooled platelet lysate (PPL, 10^8^ platelets/mL medium; Blood Donation Center Tübingen, Germany), 2mM L-glutamine (Lonza) and 1% penicillin-streptomycin (Life Technologies, Darmstadt, Germany). After 24 hours of incubation the media was discarded and replaced to remove unattached cells and media was changed twice a week. After 5–7 days, cells were removed with Accutase (PAA, Pasching Austria), counted and re-seeded in GMP expansion medium (passage 1, density 1.5x10^5^ cells per flask).

### Human Bladder Smooth Muscle Cells

Human bladder SMCs (Promocell, Heidelberg, Germany) were cultured in smooth muscle cell growth medium 2 (PromoCell) in CORNING, CellBIND surface T75 flasks (Sigma). When the cells were 90% confluent, they were split and repassaged in new tissue culture flask at a ratio of 1:4.

### Myogenic Differentiation

At passage 2 MSCs were re-seeded at a density of 1.5x10^5^ cells onto T75 cell culture flasks. When MSCs were 70% confluent, cells were cultured for 0, 3, 7, 14, 21 or 28 days in control medium: DMEM high glucose (Life Technologies), 10% FBS (Biochrom, Berlin, Germany), 1% penicillin-streptomycin solution (Life Technologies) and 1.2% fungicide (Biochrom) or smooth muscle differentiation medium (control media supplemented with 5ng/mL human recombinant PDGF-AB (Peprotech, Hamburg, Germany), 5 ng/mL recombinant human TGF-β1 (R&D Systems, Weisbaden-Nordenstadt, Germany) and 30 μM L-ascorbic acid (Sigma-Aldrich). Media were changed twice a week.

### Osteogenic Differentiation

MSCs were seeded onto 6-well plates (Greiner Bio-One, Frickenhausen, Germany) at 5x10^4^ cells/well for expansion in GMP expansion medium until they were 70% confluent. Differentiation was started by adding osteogenic induction media: low glucose DMEM high glucose (Life Technologies), 10% FBS (Biochrom), 0.1 μM dexamethasone (Fagron, Barsbüttel, Germany), 10 mM β-glycerophosphate (Sigma-Aldrich), 1% penicillin-streptomycin solution (Life Technologies) and 0.17 mM L-ascorbic acid (Sigma-Aldrich). Media were changed twice a week. At day 14, cells were fixed with ice-cold methanol and stained by von Kossa [27].

### Adipogenic Differentiation

MSCs were seeded onto 6-well plates (Greiner Bio-One) at 5x10^4^ cells/well and differentiation was started by adding adipogenic induction medium: DMEM high glucose (Life Technologies), 10% FBS (Biochrom), 1% penicillin-streptomycin solution (Life Technologies), 1 μM dexamethasone (Fagron), 0.1 mM indomethacin, 0.01 mg/mL human insulin and 0.5 mM 3-isobutylaxanthine (all from Sigma-Aldrich). Media were changed twice a week. At day 14, cells were washed and stained with Oil Red O (Sigma-Aldrich) [27].

### Quantitative RT-PCR

RNA was isolated according to the manufacturer’s protocol (RNeasy^®^ Mini Kit, Qiagen, Hilden, Germany). After extraction, cDNA was generated using Advantage^®^ RT-for-PCR-Kit (Clontech, Mountain View, California, USA). Quantitative RT-PCR was performed (Roche Diagnostics, Mannheim, Germany, LightCycler^®^ 480 II) using the LightCycler^®^ 480 SYBR Green I Master Kit to determine the mRNA expression of contractile SMC genes *ACTA2*, *TAGLN*, *CNN1* and *MYH11* (primers from Qiagen) and ion channel genes *KCNMA1* (encodes BkCa; NM_001014797; sense primer 5'-AGGAATGCATCTTGGCGTCACT-3’ and antisense primer 5'-GCGGCAGCGGTCCCTATT-3’), CACNA1C (encodes Cav1.2; NM_000719; sense primer 5'-CTGCAGGTGATGATGAGGTC-3’ and antisense primer 5'-GCGGTGTTGTTGGCGTTGTT-3’) and CACNA1H (encodes Cav3.2; NM_021098; sense primer 5'-GGAGAGCAACAAGGAGGCACG-3’; and antisense primer 5'-AGTGCACAGAGGCAACGGAG-3’) (Eurofins MWG Operon, Ebersberg, Germany) [28]. As reference genes glyceraldehyde 3-phosphate dehydrogenase (*GAPDH*, Biomol, Hamburg, Germany) and peptidylprolyl isomerase A (*PPIA*, Biomol) were used. The expression of the target genes was normalized to the reference genes. Evaluation of the results was carried out using LightCycler^®^ 480 II software release 1.5.0, method Advanced Relative Quantification.

### Immunofluorescence

MSCs at passage 1 or primary human bladder SMCs (PromoCell) were seeded onto chamber slides (SARSTEDT, Nümbrecht, Germany) at a density of 6000 cells/chamber slide in 500 μL/well GMP expansion medium. When cells were 90% confluent, medium was replaced with control or myogenic differentiation medium for MSCs, or SMC growth medium (PromoCell) for primary human bladder SMCs, respectively. After 14 days, cells were washed, fixed with ice-cold methanol and washed again. Samples were then incubated with the following antibodies diluted in PBS containing 0.1% BSA (Sigma-Aldrich): 1:100 rabbit anti-αSMA (Abcam, Cambridge, UK), 1:100 rabbit anti-transgelin (Santa Cruz Biotechnology, Heidelberg, Germany), 1:250 rabbit anti-calponin (Abcam) or 1:100 mouse anti-SM-MHC (Santa Cruz Biotechnology), followed by Cy^™^3-conjugated goat anti-rabbit or Cy^™^3-conjugated goat anti-mouse secondary antibody (dilution: 1:1000 for αSMA and transgelin; 1:500 for Calponin, 1:300 for SM-MHC; Dianova, Hamburg, Germany) for 30 min at RT. Cell nuclei were stained with DAPI, washed three times with PBS and mounted with mounting medium (Dako, Glostrup, Denmark). As a negative control cells were incubated with secondary antibody alone (data not shown). Digital microscopic images recorded in black and white and colorized to green with an exposure time of 1–1.5 s were taken with a CCD camera attached to an Axiovert M200 (Carl Zeiss, Jena, Germany) microscope at magnifications of 20x.

### Flow Cytometry for αSMA

Expression of intracellular αSMA was explored by flow cytometry (FC) as described recently [29]. To investigate MSCs *ex vivo*, the mononuclear cells were isolated from bone marrow as described above, washed twice with cold PBS, resuspended in cold PFEA puffer (PBS containing 2% FBS, 2 mM EDTA, 0.01% sodium azide), and counted. To investigate MSCs after expansion in primary cultures, the cells were harvested by mild proteolytic detachment (Accutase, PAA, Pasching Austria), washed twice with PBS resuspended in cold PFEA puffer, and counted. For FC of MSCs *ex vivo*, 1x10^6^ mononuclear cells were used, for FC of expanded MSCs, 5x10^5^ cells were used. First, cells were incubated with pre-immune serum to reduce unspecific binding of antibodies to the MSCs (Gamunex, Talecris Biotherapeutics, Research Triangle Park, NC; 1:20 in PFEA, 30 min, 4°C). The MSCs were then washed and resuspended in PFEA with one the following antibodies: CD45 (biotinylated anti-CD45, clone 2D1, R&D Systems), CD90 (PE-labelled anti-CD90, clone Thy1A1, R&D Systems), or CD271 (APC-labelled anti-CD271 clone ME20.4, Biolegend) according to the manufacturer's protocols. In the case of *ex vivo* MSCs, cells were incubated with anti-CD45 and anti-CD271 antibodies for 30 min at 4°C in the dark, washed twice, counterstained with PacificBlue-labelled strepativin conjugate as recommended by the supplier (LifeTechnologies) and washed with PFEA again. In the case of expanded MSCs, cells were incubated with the anti-CD90 for 30 min at 4°C in the dark and washed twice with PFEA. Cell membranes were then permeabilized with a mild detergent according to the manufacturer’s guidelines (cytofix/cytoperm kit, BD Bioscience). The cells were washed again with PFEA and anti-αSMA antibody (AlexaFluor488- or AlexaFluor594- labelled anti-αSMA clone 1A4, Abcam) was added to detect actin in the somata of the MSCs. FC was performed on a LSR II (BD Bioscience). To investigate MSCs *ex vivo*, viable mononuclear cells were gated (SSC/FSC), CD45^+^ cells were excluded, and expression of αSMA was recorded in the CD271^+^ fraction of mononuclear cells. To investigate αSMA in MSCs after expansion, viable cells were gated (SSC/FSC) and CD90^+^αSMA^+^ cells were recorded. The data recorded were analyzed with the DIVA (BD Biosciences) and FlowJo (FlowJo Enterprises) software programs.

### Western Blot

Lysates of undifferentiated MSCs, differentiated MSCs, human bladder tissue (positive control) and peripheral blood mononuclear cells (PBMCs) that were collected from whole blood in EDTA tubes by Ficoll density gradient centrifugation (negative control) were prepared by homogenization in modified RIPA buffer containing 1mM protease inhibitor PMSF (Oberdorla, Germany). Samples were resolved on 10% polyacrylamide (for αSMA and Calponin), 10% Bis-Tris (for Transgelin; Life Technologies) or 3–8% Tris-acetate (for SM-MHC; Life Technologies) gels. Gels were then transferred to a nitrocellulose membrane and blocked with 5% non-fat milk in 1x PBS containing 0.1% Tween20 (Merck Millipore, Darmstadt, Germany), washed and incubated overnight at 4°C with 1:1000 anti-αSMA (Abcam), 1:1500 anti-transgelin (Santa Cruz Biotechnology) or 1:1000 anti-calponin (Abcam) in 5% non-fat milk/PBS. Membranes were washed and HRP-labeled goat anti-rabbit IgG antibody (1:2000 in 5% milk/PBS) was added for 1h at RT. Samples were visualized using WesternSure Chemiluminescent Substrates (LI-COR, Lincoln, Nebraska, USA) and detected by C-DiGit^®^ Blot Scanner (LI-COR). To ensure equal loading, blots were stripped and reprobed with anti-vinculin (1:20,000 in 5% non-fat milk/PBS, Sigma-Aldrich), followed by incubation with 1:10,000 goat anti-rabbit IgG (Dako).

### Electrophysiology

Membrane currents were measured with the patch-clamp technique in the whole-cell recording configuration at RT [30]. Prior to the recordings cells were detached using Accutase (Sigma-Aldrich), stored at RT and used within 4 hours. The electrodes had a resistance between 1.5–3.5 MΩ. For MSCs the intracellular solution consisted of 20 mM KCl, 125 mM Kgluconate, 10 mM ethylene glycol-bis(b-aminoethyl ether) N, N, N’, N’-tetraacetic acid, 1 mM MgCl_2_, 10 mM Na_2_-ATP 2, HEPES, pH 7.2 and for SMCs, 145 mM KCl, 0.1 mM ethylene glycol-bis(b-aminoethyl ether) N, N, N’, N’-tetraacetic acid, 2mM MgCl, 2 mM Na_2_-ATP, 10 mM HEPES, pH 7.2. The extracellular solution consisted of 130 mM NaCl for MSCs and 115 mM for SMCs, containing 2.5 mM KCl, 1 mM MgCl_2_, 10 mM BaCl_2_, 10 mM HEPES, 10 mM glucose, and 10 mM tetraethylammonium chloride, pH 7.35. Tetrodotoxin citrate (Sigma), ranolazine hydrochloride (Sigma-Aldrich) and pro-toxin II (Tocris, Magdeburg, Germany) were prepared as stocks and frozen until use. Cells were held in voltage clamp mode by an EPC10 amplifier (HEKA, Lambrecht, Germany), and currents were filtered at 2.9 kHz using the internal Bessel filter, digitized and stored at 50 kHz. Cells were held at -70mV between the different recordings. Experiments were analyzed using the Processing and Analysis Tool developed by J. Bergsman and self-written macros in Igor Pro 6.35 (Wavemetrics, Lake Oswego, Oregon, USA).

### Calcium Imaging

Global Ca^2+^ transients were measured using fura-2 (fura-2-AM, 0.4–0.75 μM, from a stock of 1 mM in DMSO/20% pluronic) for 30 min at RT. Prior to recording, the loading solution was exchanged with extracellular solution. For depolarization of the cells, the K^+^ concentration was quickly raised to 15 mM by adding a stock solution of KCl. CdCl_2_ (Sigma-Aldrich) was added to block the Ca^2+^ channels at the concentrations indicated. Coverslips with cells were mounted in a custom made chamber on the stage of an inverted microscope (iMic, Till Photonics; Munich, Germany). The system was comprised of a video camera (SensiCam, PCO imaging; Kelheim, Germany) and a monochromator for excitation (Polychrome IV, Till Photonics). Imaging was performed by exciting the cells alternating between the Ca^2+^-dependent wavelength (380nm) and the Ca^2+^-independent isosbestic excitation wavelength of 355 nm and recording the fluorescence signal (>440 nm).

### Contractility Assay

Potassium chloride (KCl)-induced contractile activity of the differentiated cells was measured using a modification of the method described in [31]. For generating fibrin hydrogels, the following working solutions were produced: fibrinogen (Sigma-Aldrich; 2mg/mL DMEM) and bovine thrombin (Sigma-Aldrich; 5U/mL PBS). Of these working solutions, 500μl fibrinogen per ml hydrogel and 20μl thrombin per ml hydrogel were mixed with 380μl low glucose DMEM and 100μl FBS for the generation of hydrogels (1.9 cm^2^). Prior to polymerization, this suspension (500μl/well) was pipetted into 24-well tissue culture treated plates (Greiner bio-one, Frickenhausen, Germany). After 30 min, during which the hydrogels polymerized at 37°C, 2500 MSCs or 1500 SMCs were added onto the hydrogel surface in 500μl culture medium. The cells were incubated on top of these hydrogel surfaces in their respective media at 37°C for 24h. To initiate the contraction experiments, media was removed and 0.5 mL/well of physiological saline solution (PSS composed of 99.1% deionized water and 0.9% NaCl) and green Calcein AM-fluorescence dye (0.6:1000) were gently added until the hydrogels were completely submerged. The cells were incubated for 30 minutes in PSS to normalize prior to contraction. PSS was removed and 0.5 mL/well potassium physiological saline solution (KPSS composed of 99.1% deionized water and 0.9% KCl) was gently pipetted into the plate until the hydrogels were completely submerged. Digital images were recorded with a confocal laser scanning microscope (Zeiss LSM 510, Axio Vision 4.8) exactly 10 seconds and 3 minutes after the addition of KPSS. To ensure comparability across all recorded images, a similar magnification (10x) and a similar size of the recorded image field (1388 x 1040 pixel) were used throughout these experiments. The images of 70 MSCs in control media (from 3 donors on 24 hydrogels), 58 MSCs in differentiation media (from 3 donors on 24 hydrogels), and 62 SMCs (on 32 hydrogels) were selected for further analysis based on the criteria that they were i) in focus and ii) that the entire cell was within the image frame. The images of each individual cell at the time points of 10 seconds and 3 minutes were contrast-enhanced by 5% and side-by-side manually thresholded with ImageJ (NIH, http://imagej.nih.gov/ij) while comparing the resulting thresholds with the originally recorded images. Cellular length was measured using the commands “set measurements” and “analyze particles”. Contraction was determined by the change in length observed between the time points 10 seconds and 3 minutes and was expressed as percentage of the initial length at 10 seconds.

### Statistics

Statistical analyses were performed using SigmaPlot 11.0 using a nonparametric Kruskal-Wallis test followed by a post hoc Dunn-Holland-Wolfe test or a Wilcoxon Signed Rank Test. When only two groups were compared a paired t-test was performed. P values < 0.05 were considered significant.

## Results

### Expression of Contractile SMC Marker Genes Increases within 1–2 Weeks of Myogenic Differentiation

**Fig 1:**
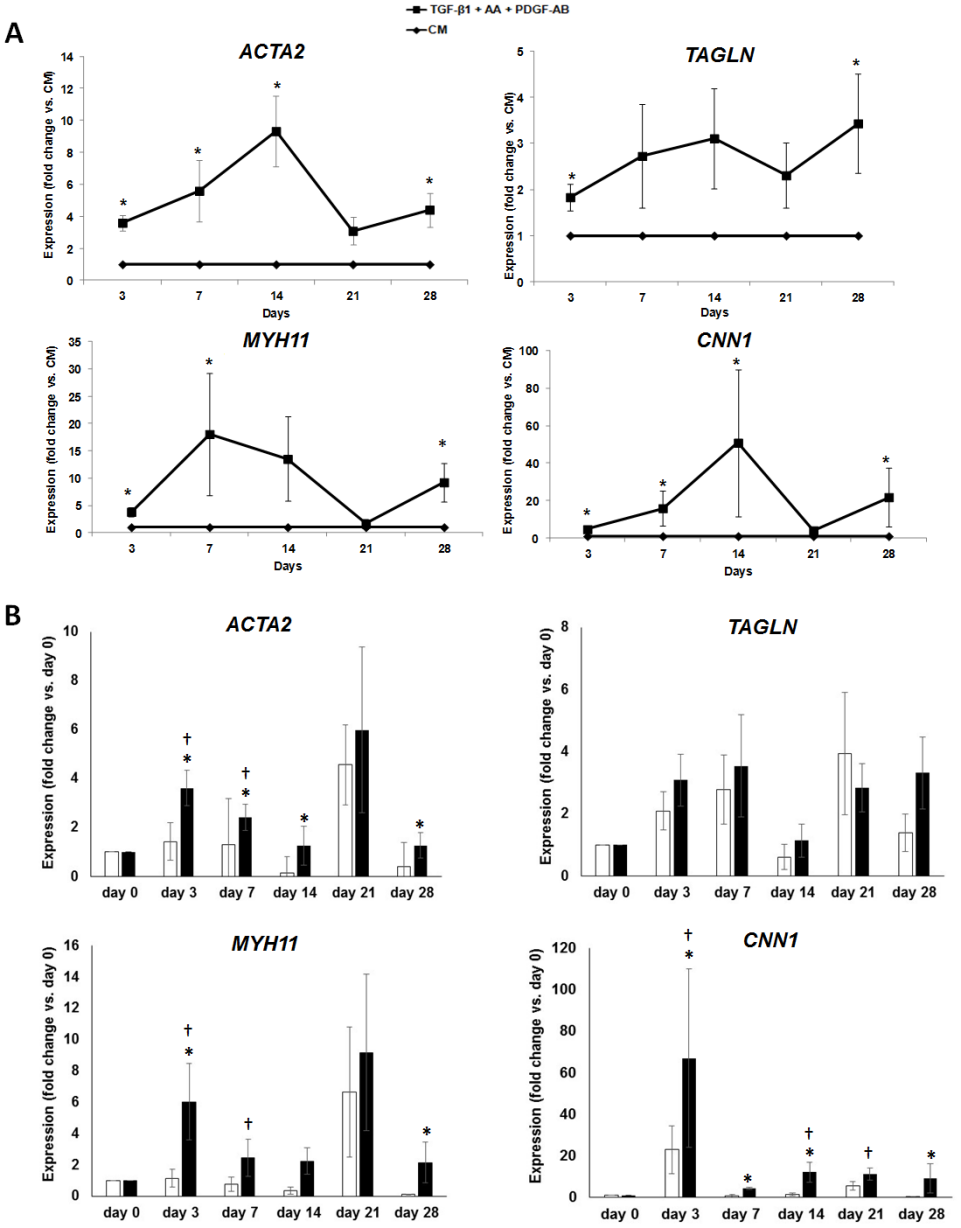
Expression levels of contractile SMC-specific genes. MSCs were expanded in GMP expansion medium until they were 70% confluent and at passage 2 treated with control medium (CM) or SMC differentiation medium (5 ng/mL human TGF-β1, 5 ng/mL human PDGF-AB and 30 μM ascorbic acid) for 0, 3, 7, 14, 21 and 28 days. Differentiation was analyzed by qRT-PCR. Transcript levels were calculated relative to *GAPDH* and *PPIA*. (A) Data was calculated relative to CM for that time point. * p<0.05 compared to CM at the respective day of differentiation. (B) Data was calculated relative to day 0, the starting point of differentiation. * p<0.05 compared to CM at the respective day of differentiation; ^†^ p<0.05 compared to day 0. *n* = 6–8. Error bars indicate SEM.

MSCs were first expanded in GMP expansion medium containing human plasma and pooled platelet lysate and then differentiated using a combination of TGF-β1, ascorbic acid and PDGF-AB. The gene expression of contractile myogenic markers was measured by qRT-PCR and analysed and expressed as fold increase with respect to control medium for that particular day of differentiation (Fig 1A) or as fold increase with respect to day 0 (Fig 1B). When compared to control medium, differentiation of MSCs resulted in a significant increase in *ACTA2* at days 3, 7, 14 and 28; *TAGLN* at days 3 and 28; *MYH11* at days 3, 7 and 28; and *CNN1* at days 3, 7, 14 and 28 (Fig 1A). In agreement with these results, when the expression of these markers were normalized with respect to day 0, the starting point of differentiation, there was a significant increase in *ACTA2* at days 3, 7, 14 and 28; *MYH11* at days 3 and 28; and *CNN1* at days 3, 7, 14 and 28 expression compared to control media (Fig 1B). In contrast, using this method of analysis *TAGLN* was not significantly increased compared to control media at any of the days investigated or compared to day 0 of differentiation. However, compared to day 0 of differentiation, *ACTA2* and *MYH11* were significantly increased at days 3 and 7, while *CNN1* significantly increased at days 3, 14 and 21. This data demonstrates that myogenic differentiation already increases the expression of contractile marker genes *ACTA2*, *MYH11* and *CNN1* within 1–2 weeks of differentiation.

### Myogenic Differentiation Results in Increased Expression of Contractile SMC Marker Proteins

**Fig 2:**
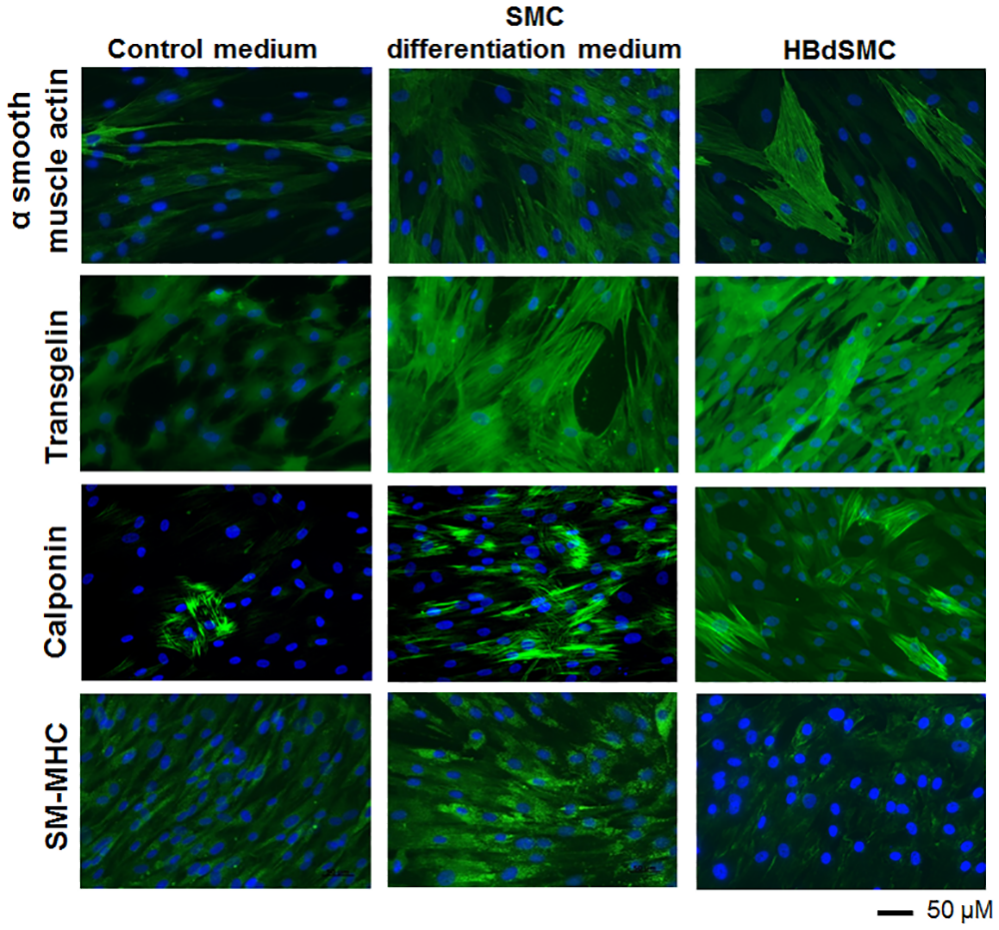
Expression of contractile SMC-specific proteins analyzed by immunofluorescence. MSCs were expanded in GMP expansion medium until they were 70% confluent and at passage 2 treated with control medium or SMC differentiation medium for 14 days, fixed and then analyzed by immunofluorescence for expression of αSMA, transgelin, calponin and SM-MHC. Primary human bladder smooth muscle cells (HBdSMC) served as the positive control. Nuclei were stained with DAPI. Magnification 20x. Representative of *n* = 3.

We next compared the protein expression of these contractile SMC markers in primary human bladder SMCs compared to MSCs cultured in control or myogenic differentiation media for 14 days. Despite that some protein expression was already evident in MSCs in control media, differentiation of MSCs resulted in an increase in protein levels of alpha smooth muscle actin (αSMA), transgelin, calponin and smooth muscle myosin heavy chain (SM-MHC) protein levels at day 14 following myogenic differentiation as shown by immunofluorescent staining (Fig 2). Moreover, the expression of these markers in differentiated MSCs were comparable to primary human bladder SMCs.

**Fig 3:**
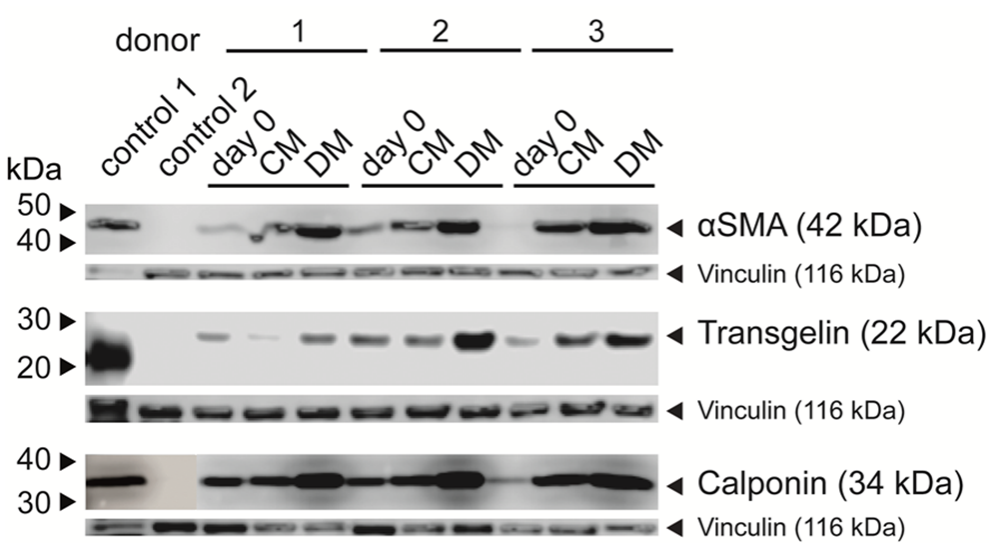
Expression of contractile SMC-specific proteins analyzed by Western blot. MSCs from three donors were treated with control medium (CM) or SMC differentiation medium (DM) for 0 or 14 days and the expression pattern of αSMA, transgelin and calponin was compared to the positive control (control 1: bladder tissue) and the negative control (control 2: PBMCs) by Western blot. Representative of *n* = 3.

To further confirm that the protein expression of myogenic markers was increased following differentiation using the TGF-β1, ascorbic acid and PDGF-AB cocktail, Western blots were performed on MSCs after expansion and after culturing MSCs in control media or differentiation media for 2 weeks. Western blotting using lysates of human bladder tissue (positive control; control 1) and PBMCs (negative control; control 2) compared to three different MSC donors confirmed a baseline expression of myogenic markers after expansion (day 0) and showed that in some cases culturing MSCs in control media (CM) increased expression of myogenic markers (Fig 3), in agreement qRT-PCR data (Fig 1B). Even though there was a baseline expression of these myogenic markers, we still observed an increase in expression of αSMA, transgelin, and calponin following myogenic differentiation (DM) compared to day 0 and compared to MSCs cultured in control media (CM). Variable results were observed with expression of SM-MHC, in some cases an increase was observed and in other cases not (data not shown).

### After MSCs Expansion αSMA Is Expressed in Some of the MSCs but Can Be further Elevated by Myogenic Differentiation

**Fig 4:**
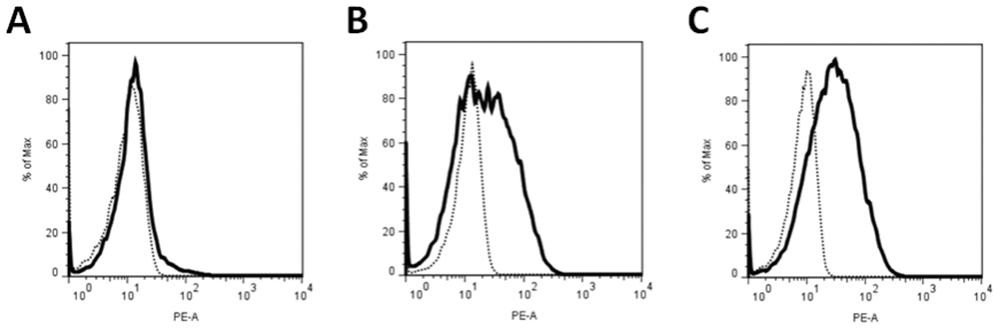
Expression levels of αSMA *ex vivo*, after MSCs expansion and myogenic differentiation *in vitro*. (A) Mononuclear cells were directly isolated from bone marrow *ex vivo* and assessed for expression of αSMA by flow cytometry. Viable cells were gated (SSC/FSC), CD45^+^ cells were excluded, and expression of αSMA was recorded in the cytoplasm of the CD45^-^CD271^+^ fraction of the mononuclear cells. The histogram represents αSMA in the cytoplasm of CD271^+^ cells (solid line) compared to the unstained controls (dotted line). (B) MSCs were expanded in GMP-compliant expansion medium and then assessed for expression of αSMA in the cytoplasm of the cells. Viable cells were gated (SSC/FSC) and αSMA^+^ cells were recorded (solid line). The dotted histogram represents the unstained controls. The broad profile of the histogram indicates that a large portion of MSCs express αSMA after expansion and prior to induction of differentiation (27% of MSCs were positive, MFI of 38). (C) After expansion in GMP-compliant expansion medium MSCs were differentiated for 14 days. Then expression of αSMA in the cytoplasm of differentiating MSCs was explored (solid line). The histogram indicates that more cells contain αSMA after differentiation (27% of MSCs were positive, MFI of 42, right panel). The dotted lines represent unstained controls.

As the GMP-compliant expansion medium was enriched with PPL, we hypothesized that TGFβ1 and PDGF in the medium induced the base line expression of smooth muscle cell markers αSMA, transgelin or calponin observed in MSCs prior to differentiation (Fig 4). To test this hypothesis we investigated the expression of the early myogenic marker αSMA [31] in MSCs before and after expansion of MSCs and after myogenic differentiation. To discriminate MSCs from other cells in the samples *ex vivo*, CD45^+^ hematopoietic cells were excluded and expression of αSMA was recorded in the CD271^+^ fraction of the mononuclear cells [32]. Flow cytometry results showed that expression of αSMA was not detected the CD45^-^CD271^+^ MSCs *ex vivo* (Fig 4A). In contrast, the CD45^-^CD271^-^ mononuclear cells from bone marrow clearly contained αSMA (data not shown). However, after expansion of the cells, levels of intracellular αSMA increased in some of the MSCs (Fig 4B) suggesting that the expression of this marker was elevated by growth factors within the GMP expansion medium. This is not surprising since PLL is known to supply a wide variety of growth factors [33], including TGFβ1 and PDGF employed for myogenesis in many studies [17,22,23,26] and here (Figs 1–3). But not all MSCs expressed αSMA at elevated levels and some remained αSMA^low^ as indicated by the broad histogram (Fig 4B). After incubating the MSCs in differentiation medium the mean fluorescence intensity of αSMA expression shifted to a slightly higher signal intensity (Fig 4C). This experiment confirmed our hypothesis that only a few MSCs express αSMA *ex vivo*, but after expansion under GMP-compliant conditions a considerable number of MSCs expresses αSMA. Moreover this showed that the expression of αSMA can be further elevated by myogenic differentiation media (Fig 4), in agreement with the results shown in Figs 2 and 3.

### Osteogenesis and Adipogenesis Does Not Occur in Myogenic Differentiation Medium

**Fig 5:**
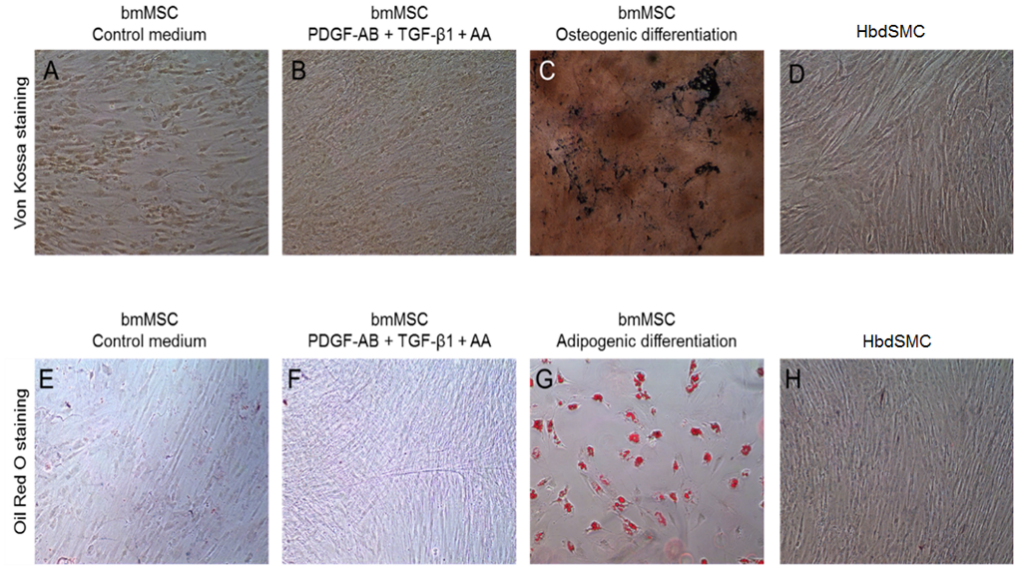
Determination of osteogenic and adipogenic differentiation. MSCs were cultured in control medium, SMC differentiation medium, osteogenic differentiation medium or adipogenic differentiation medium as indicated for 14 days. HbdSMCs were cultured in SMC medium (smooth muscle cell growth medium 2). Osteogenic differentiation was assessed by Von Kossa staining (A-D) and adipogenic differentiation by Oil Red O staining (E-H). Magnification 20x. Representative of *n* = 3.

Since we have shown that bone marrow-derived MSCs have a higher osteogenic potential than placenta-derived MSCs [29,34,35] we wanted to determine if MSCs cultured in the myogenic medium containing TGF-β1, ascorbic acid and PDGF-AB tended to differentiate towards an osteogenic lineage. As expected, MSCs cultured in osteogenic differentiation medium resulted in strong von Kossa staining (Fig 5C). In contrast, after 2 weeks of differentiation MSCs cultured in control medium or myogenic medium did not differentiate into osteoblasts as visualized by von Kossa staining (Fig 5A and 5B, respectively). Similarly, primary human bladder SMCs stained with von Kossa chemistry did not show signs of osteogenesis when expanded in smooth muscle cell growth medium 2 (Fig 5D). It should be noted that in rare instances we observed spots of von Kossa staining following myogenic differentiation of MSCs, but in these sporadic instances there were only minor precipitations (positive von Kossa staining) and on only a few of the cells (data not shown). This may indicate remaining alkaline phosphatase activities in MSCs which did not undergo entry into the myogenic lineage.

Similarly we also investigated whether any adipogenesis occurred following myogenic differentiation. As expected, adipocytes were only observed in MSCs cultured in adipogenic differentiation medium (Fig 5G). No lipid-containing vesicles were observed in MSCs cultured in control or myogenic medium (Fig 5E and 5F, respectively). Similarly, primary human bladder SMCs stained with Oil Red O did not show signs of adipogenesis when expanded in smooth muscle cell growth medium 2 (Fig 5H). These results show that myogenic differentiation does not lead to osteogenesis or adipogenesis.

### Voltage-gated Na^+^ Current Levels Were Sensitive to a Selective Sodium Channel Blocker that Blocks Neuromuscular Junctions

**Fig 6:**
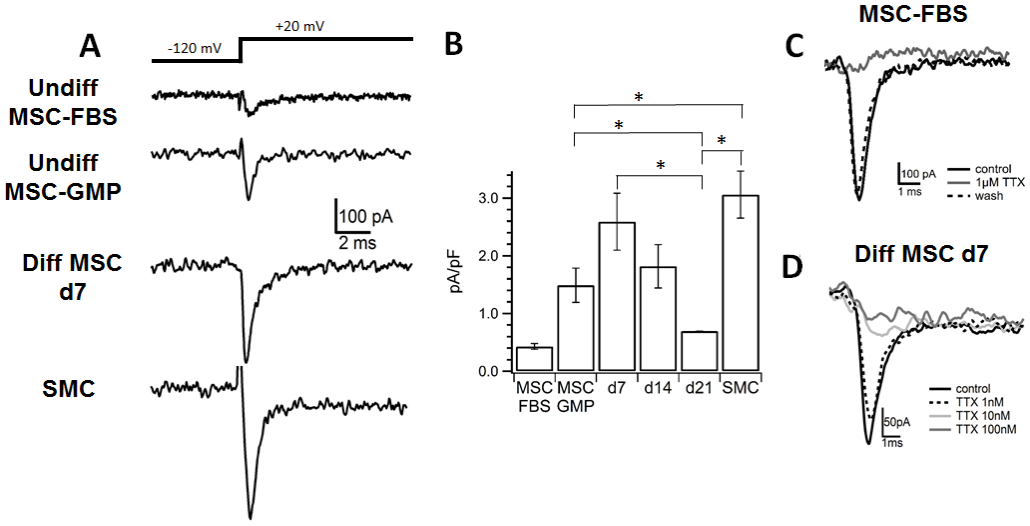
Levels of voltage-activated Na^+^ currents and Na^+^ channels. (A) Voltage-activated Na^+^ currents were elicited by a voltage step from -120 mV to +20 mV. MSCs: trace from undifferentiated MSCs in control medium (undiff MSC-FBS) and GMP expansion medium (MSC-GMP); d7: trace from MSCs after 7 days in myogenic differentiation medium; SMC: trace from primary bladder SMCs. Capacitive transient is blanked for better visualization. (B) Na^+^ current density for undifferentiated MSCs in control medium (MSC FBS) and GMP medium (MSC GMP), as well as MSCs differentiated for 7, 14 or 21 days and SMCs, respectively. *n* = 10–20. * p<0.05. Error bars indicate SEM. (C) Effect of TTX on Na^+^ currents in undifferentiated MSCs (here: MSC in control medium). Superposition of Na^+^ currents elicited at +20mV. Na^+^ channels could be blocked by the specific Na^+^ channel inhibitor TTX. (D) Effect of TTX on Na^+^ currents in MSCs that were differentiated for 7 days. Superposition of Na^+^ currents elicited at +20mV. TTX blocks the current concentration-dependently.

Since ion channels play an important role in regulating the contraction of urinary bladder smooth muscle [14], electrophysiological experiments were performed on undifferentiated MSCs, differentiating MSCs and primary human bladder SMCs to assess the functional properties of the differentiating MSCs. When MSC from 2 patients were cultivated in FBS containing control medium (MSC-FBS), in both sources 40% of the cells investigated (4 out of 10) expressed a Na^+^ current (Fig 6A top), confirmed by the fact that this current could be blocked by the specific Na+ channel inhibitor tetrodotoxin (1 μM; TTX; a selective sodium channel blocker; Fig 6C). The current density normalized to the cell surface was small with 0.43 ± 0.05 pA (Fig 6B, obtained at a holding potential of -20 mV). On the other hand, MSCs expanded in GMP expansion medium (MSC-GMP) only rarely expressed functional Na^+^ channels. In fact only in cultures from 3 donors out of 18, MSC could be found, expressing Na^+^ current densities of 1.4±0.2 pA/pF (n = 10). Hence voltage-activated Na^+^ channels were rarely found in MSC-GMP.

In contrast, after myogenic differentiation Na^+^ currents were found in 40–60% of all cells investigated, depending on the time of differentiation. The current size in those cells peaked to 2.5±0.5 pA/pF at day 7 (n = 5; Fig 6A middle) following myogenic differentiation. Moreover, differentiated cells were similar to primary bladder SMCs (n = 20; Fig 6A bottom). Hence the Na^+^ current density of 3.1 ± 0.9 pA/pF in primary SMCs was not significantly different from those obtained at day 7 of differentiation. Interestingly, with progressing differentiation time the amplitude decreased again (Fig 6B). Moreover, the Na^+^ channels of both cell types, myogenically differentiated cells and bladder SMCs, were sensitive to the Na^+^ specific blocker TTX (Fig 6C shown for differentiated cells), with a half maximal inhibition (IC_50_) of 20 nM and 40 nM for day 7 differentiated cells and SMCs, respectively, indicating the functional role of differentiated MSCs comparable to bladder SMCs.

### The Voltage-Gated Sodium Channel Is of the Na_v_1.4 Subtype

**Fig 7:**
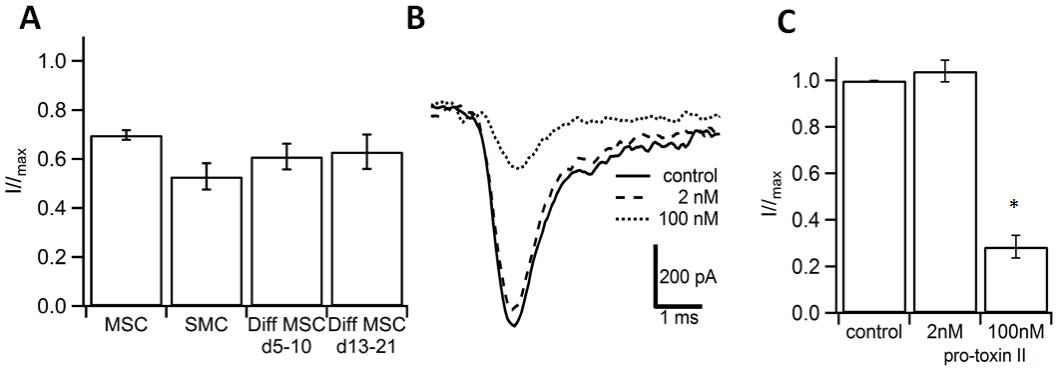
Blockage of voltage-gated Na^+^ channel subtypes. (A) Application of 100nM ranolazine reduced the peak amplitude of voltage-activated Na^+^ channels [(*n* = 3 for undifferentiated MSCs (cultured in GMP expansion medium), *n* = 4 for SMCs, *n* = 5 for MSCs that were differentiated for 5–10 days and *n* = 7 for MSCs differentiated for 13–21 days (Diff MSC)] compared to the respective control (= 1.0, not shown). (B) Application of pro-toxin II inhibited voltage-gated Na^+^ channels in SMCs. Superposition of single current traces obtained in control (bold), at 2nM (dashed) and 100nM (dotted) from one donor, respectively. (C) Summary plot of current inhibition by pro-toxin II. Data obtained from n = 4 experiments. * p<0.05. Error bars indicate SEM.

Ranolazine is a drug known to partially block Na_v_1.4 and Na_v_1.7 voltage-gated sodium channel subtypes [36]. Application at a concentration of 100 μM blocked voltage-activated Na^+^ currents by approximately 30% for undifferentiated MSCs (n = 3), 40% for MSCs that were differentiated for 5–10 days (n = 5) or 13–21 days (n = 7) and 50% for SMCs (n = 4) (Fig 7A). Pro-toxin II, a toxin from the tarantula spider, inhibited Na_v_1.4 with an IC_50_ of 40nM. Na_v_1.7 inhibition, on the other hand, was more sensitive with an IC_50_ of 0.3 nM [37] (Fig 7B). In SMCs, pro-toxin II failed to inhibit the Na^+^ current at a concentration of 2 nM, whereas 100 nM suppressed the current by 79% (n = 4; Fig 7C) indicating that the main component of this current was mainly mediated by the Na_v_1.4 channel.

### Differentiated MSCs Respond by Increasing Cytosolic Ca^2+^

**Fig 8:**
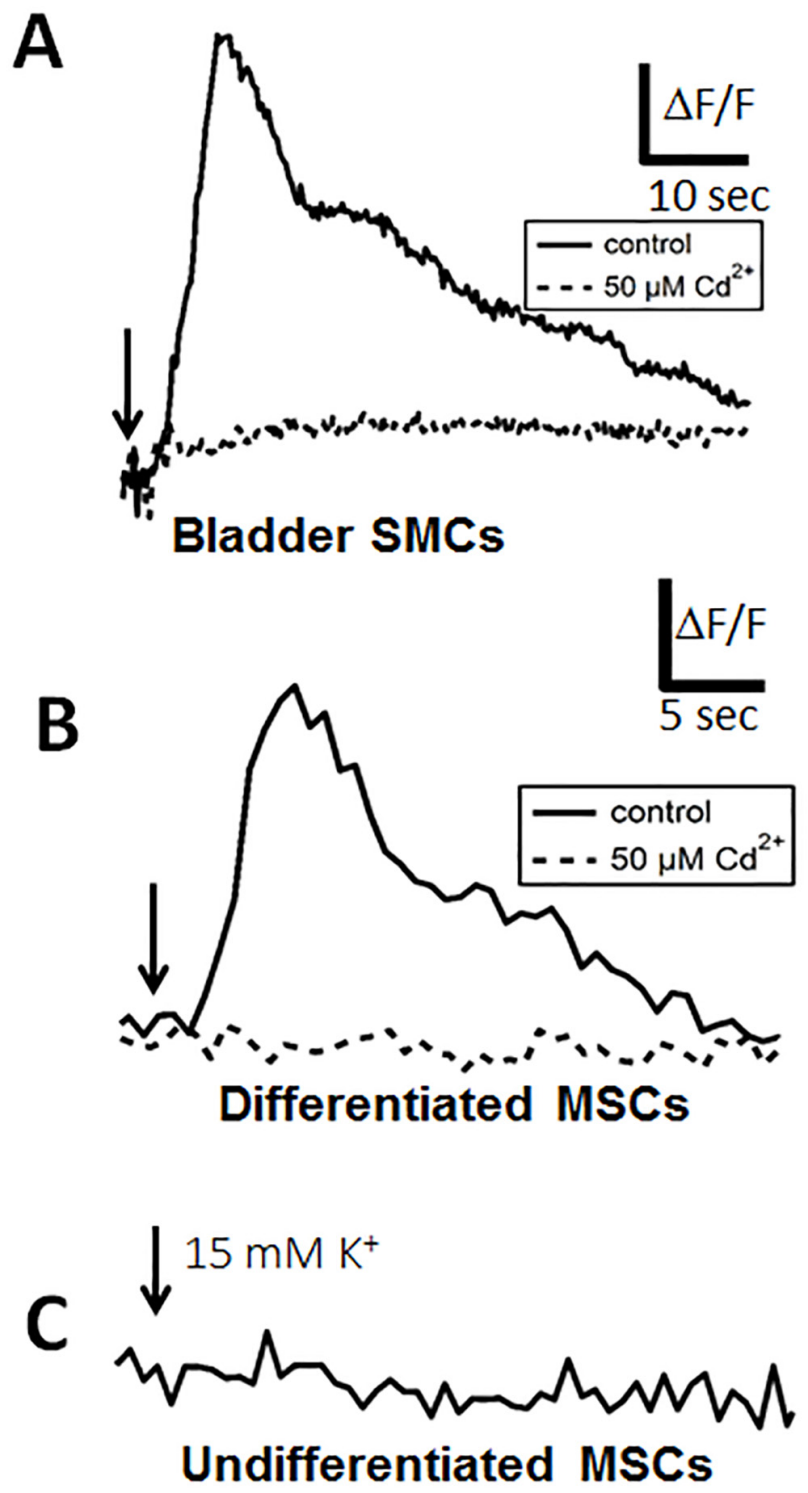
Levels of intracellular Ca^2+^. Ca^2+^ imaging of (A) bladder SMCs and (B) differentiated MSCs (d7). K^+^-induced depolarization increased the intracellular Ca^2+^ content (black trace). Depolarization in the presence of 50 μM Cd^2+^ prevented the Ca^2+^ increase (dashed trace). (C) In undifferentiated MSCs (expanded in GMP expansion medium) no transient increase in cytosolic Ca^2+^ was observed in response to K+ induced depolarization. Arrow indicates time point in which 15 mM K^+^ was added to the bath solution.

SMCs express voltage-gated Ca^2+^ channels, and like all muscle cells bladder smooth muscle use Ca^2+^ as a trigger for contraction by mediating membrane potential dependent Ca^2+^ influx through channels in the plasma membrane. This in turn triggers Ca^2+^ release from internal stores like the sarcoplasmatic reticulum and mediates contraction of the cells [13]. In order to investigate if elevated intracellular Ca^2+^ concentrations can be transiently initiated, myogenically differentiated cells and bladder SMCs were depolarized by transiently elevating the extracellular K^+^ concentration by 15mM, resulting in a cell depolarization beyond the threshold for activation of voltage-gated Ca^2+^ channels. Like bladder SMCs (Fig 8A), myogenically differentiated MSCs expressed a pronounced elevation in the cytosolic Ca^2+^ in all cells investigated (n = 26 from 2 donors; Fig 8B), whereas no increase in the intracellular Ca^2+^ concentration was visible in undifferentiated MSCs (n = 45 from 3 donors; Fig 8C). This was in line with the finding, that in neither MSC-GMP nor MSC-FBS Ca^2+^ currents could be observed (data not shown), which is the key requirement for the sarcoplasmatic Ca^2+^ release [13]. The elevation in bladder and differentiated MSCs was suppressed in the presence of 50 μM Cd^2+^, a potent inhibitor of voltage-gated Ca^2+^ channels (n = 5 from 1 donor; Fig 8A and 8B, respectively). This is an important finding since an increase in intracellular Ca^2+^ concentration is a key process required for the activation of contraction in bladder smooth muscle [13].

### Differentiated MSCs and Bladder SMCs Express Big-Conductance Ca^2+^-activated K^+^ Ion Channels and L- and T-Type Ca^2+^ Ion Channels

**Fig 9:**
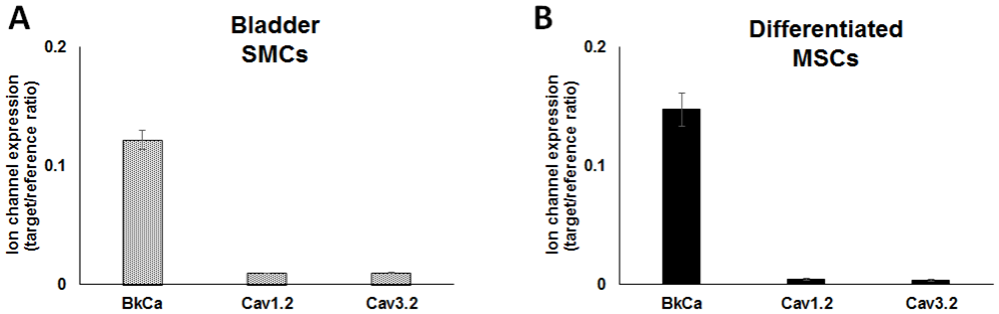
Expression levels of potassium and L- and T-type calcium ion channels. (A) Bladder SMCs were compared to (B) MSCs treated with SMC differentiation medium for 7 days for expression of ion channels. Transcript levels of *KCNMA1* (potassium ion channel), *CACNA1C* (Ca_v_1.2 L-type Ca^2+^ channel) and *CACNA1H* (Ca_v_3.2 T-type Ca^2+^ channel) were measured by qRT-PCR and expressed as target/reference ratio relative to *GAPDH* and *PPIA*. *n* = 3. Error bars indicate SEM.

To identify additional ion channels expressed in differentiated MSCs and bladder SMCs, we performed qRT-PCR for the gene expression of potassium and calcium ion channels that regulate contraction of bladder SMCs [38–40]. Like bladder SMCs, differentiated MSCs (day 7) showed a similar profile, with no significant differences between the two groups, in the expression of *KCNMA1*, *CACNA1C* and *CACNA1H* (Fig 9A and 9B, respectively). Hence bladder SMCs and differentiated MSCs expressed high levels of *KCNMA1* that encodes BK_Ca_ channels, potassium channels characterized by their large conductance for potassium (K^+^) ions through cell membranes and lower levels of *CACNA1C* and *CACNA1H* which encode for the Ca_v_1.2 L-type Ca^2+^ channels and the Ca_v_3.2 T-type Ca^2+^ channels, respectively [39,40].

### Differentiated MSCs and Bladder SMCs Contract in Response to K^+^

**Fig 10:**
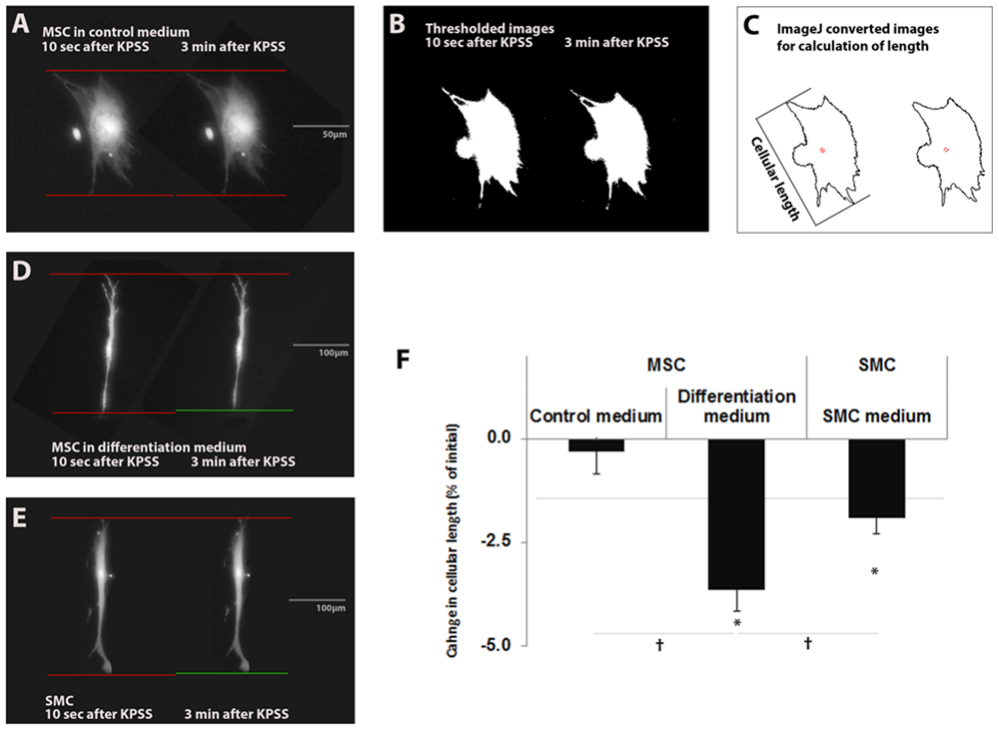
Contraction of differentiated MSCs and bladder SMCs. Contraction was measured by quantifying changes in cellular length. MSCs of three different donors were cultured in control medium or differentiation medium for 7 days and compared to bladder SMCs. To illustrate the process of determining cellular length, representative images of Calcein-stained MSCs in control medium 10 seconds and 3 minutes after KPSS addition are shown in (A). The red lines are reference lines for assessing cellular dimensions at each time point. The green line indicates a change in the cell length. The images generated through manual thresholding are illustrated in (B). Note that the threshold dimensions (highlighted in white) correspond exactly with the original cellular dimensions in (A). (C) Automated image processing for measuring cellular length resulted in images highlighting cellular outlines, upon which the measurement of cellular length was based. Representative images of 70 MSCs in control medium, of 58 MSCs that were differentiated for 7 days and of 62 bladder SMCs as shown in A, D and E, respectively. A decrease in cellular length was emphasized with red and green lines depicting cellular dimensional changes (D and E). Quantification of these changes in cellular length are depicted in (F) and expressed as change in cellular length (% of initial). Error bars indicate SEM. * p<0.05 comparing cell length at 10 seconds vs. 3 minutes after KPSS addition show that both differentiated MSCs and SMCs, but not MSCs in control medium, exhibited a significant decrease in cellular length. ^†^ p<0.05 compared to control medium and bladder SMCs show that MSCs in differentiation medium exhibited a significantly larger change in cellular length, compared to MSCs in control medium and to SMCs.

Contraction is commonly assessed by measuring changes in hydrogel dimensions. However our approach was to directly measure the contraction of the individual cells on top of the hydrogels, and not the effects of cellular contraction on hydrogel dimensions. To examine whether differentiated MSCs were able to contract, they were subjected to the effects of KCl (potassium physiological saline solution or KPSS). MSCs were cultured in control or differentiation medium for 7 days and compared to bladder SMCs. When comparing MSCs in control medium 10 seconds and 3 minutes after KPSS addition, no change in cellular length occurred (Fig 10A). In contrast, when comparing MSCs in differentiation medium 10 seconds and 3 minutes after KPSS addition, a decrease in cellular length occurred (Fig 10D). When comparing SMCs in their respective medium 10 seconds and 3 minutes after KPSS addition, a decrease in cellular length also occurred (Fig 10E). Quantification of the changes in cellular length are depicted in Fig 10F. Both MSCs in differentiation medium and SMCs, but not MSCs in control medium, exhibited a significant decrease in cellular length when comparing their length at 10 seconds and 3 minutes after KPSS addition. Moreover, differentiated MSCs exhibited a significantly larger change in cellular length (-3.57 ± 0.52% of the initial cell length), compared to MSCs that were cultured in control medium (-0.31 ± 0.56%) and to SMCs (-1.86 ± 0.37%). These values depict the average contraction of the cells investigated. A small proportion of the differentiated MSCs and bladder SMCs did not contract. Hence 10 out of a total of 59 cells investigated for differentiated MSCs and 11 out of a total of 63 bladder cells investigated did not show a decrease in cell length suggesting that approximately 17% of both cell types did not exhibit the contractile phenotype. However it is noteworthy to state that a maximum contraction of 14.43% for differentiated cells and 12.91% for bladder SMCs was observed in some cells. Based on these results we conclude that KPSS treatment induced the contraction of differentiated MSCs, like bladder SMCs. Collectively, these results can be seen as an important step towards the successful differentiation of MSCs into smooth muscle-like cells with electrophysiological competence comparable to bladder SMCs.

## Discussion

The main finding of this study is that myogenically differentiating MSCs display marker gene expression and acquire electrophysiological competence comparable to bladder smooth muscle cells. While undifferentiated MSCs have been proposed to repair smooth muscle tissue of the urinary tract such as the bladder or urethral sphincter [9,41], this method depends on the surrounding cell- and tissue-based signals to induce the progenitor cells to differentiate into SMCs and some studies have shown that only a small portion of the undifferentiated MSCs differentiate into smooth muscle [10,41]. MSCs could alternatively be pre-differentiated *in vitro* prior to implantation which may allow control over the signals required for SMC differentiation. In this study we measured the effects of GMP expansion on myogenic differentiation using a cocktail of TGF-β1, ascorbic acid and PDGF-AB and analyzed the expression of contractile myogenic markers in relation to the electrophysiological parameters to assess the functional role of the differentiated MSCs.

Specifically we show that within 1–2 weeks of myogenic differentiation differentiating MSCs significantly expressed alpha smooth muscle actin, transgelin, calponin, smooth muscle myosin heavy chain, large conductance Ca^2+^-activated K^+^ channel BK_Ca_ channels, Ca_v_1.2 L-type Ca^2+^ channels and Ca_v_3.2 T-type Ca^2+^ channels according to qRT-PCR and/or immunofluorescence and Western blot. Moreover we show that differentiated cells exhibit functional electrophysiological competence comparable to bladder SMCs which is important since ion channels are essential in regulating the contractile proteins involved in physiological contraction and are responsible for smooth muscle function by regulating the membrane potential and excitability of SMCs [13,31]. Hence, any changes in membrane potential can coordinate a contraction across large distances through gap junctions that allow ionic or electrical coupling of cells [13].

The expression of the myogenic markers observed in our study, in part, confirmed data from other groups who investigated differentiation of human bone marrow-derived MSCs into SMCs [17,18,22]. All of these phenotypic markers regulate or are directly involved in contraction of SMCs. For example αSMA, an early differentiation marker [13,31,42], forms the thin filament of the classical contractile apparatus [43]. Transgelin, an early marker of smooth muscle differentiation [44] and calponin, an intermediate marker [13,31,42], binds to actin to regulate SMC contraction [45]. Functionally, SM-MHC appears to be the most mature SMC marker [13,31,42] since it functions as an ATPase to generate force along actin filaments [46]. In contrast to a recent study [17], in our study *MYH11* was significantly increased on the gene expression level and in agreement with their results [17] SM-MHC was only slightly increased on the protein level. The differences that we observed may be due to the differences in tissue culture conditions such as the use of human products in the GMP expansion medium that we used (vs. FBS), the tissue culture conditions or the amount of TGF-β1 in the culture medium since we used double the concentration of TGF-β1 (5 ng/ml) [17].

Because differentiation markers can also change in control medium (which contained FBS and hence growth factors) as shown in Fig 1B and Figs 2–4, we also analyzed the data to account for this. However, even when comparing the fold change in gene expression relative to day 0, differentiated cells expressed significantly more *ACTA2*, *CNN1* and the late myogenic differentiation marker *MYH11*. Moreover the number of αSMA^pos^ cells was further elevated by myogenic differentiation medium and differentiation of MSCs resulted in an increase in αSMA, transgelin, and SM-MHC proteins. Although cultivating cells in control medium seemed to partly initiate spontaneous differentiation of the MSCs, as demonstrated by a Na^+^ current in approximately 40% of the cells recorded compared to 17% in GMP-cultured MSCs, the Na+ current density was only 1/3 of the current size found in GMP-cultured MSCs and no Ca^2+^ currents were observed in control medium- or GMP-cultured MSCs. Moreover, cells cultured in control medium did not contract in response to K^+^.

In contrast, differentiated MSCs, like bladder SMCs, expressed functional Na^+^ channels. In differentiated MSCs the current density peaked at day 7 to similar levels as in bladder SMCs. Pharmacological profiling indicated that the main Na^+^ channel subtype observed in this study was Na_v_1.4. This is in line with another study showing that the Na_v_1.4 channel is expressed in SMCs of the stomach fundus and ureter [47], and this particular channel subtype has been proposed to modulate contraction through a mechanism involving reverse mode Na^+^—Ca^2+^ exchange [48–50].

Also here, unlike undifferentiated MSCs, both differentiated MSCs and SMC reacted with transiently elevated cytosolic Ca^2+^ to KCl-induced depolarization, which is the key upstream process of contraction. KCl is commonly used as a stimulus to activate SMCs by a highly reproducible mechanism involving activation of voltage-operated Ca^2+^ channels that leads to increases in cytosolic Ca^2+^, Ca^2+^-calmodulin-dependent myosin light chain kinase activation, MLC phosphorylation and finally contraction [51]. Hence physiological depolarization during action potentials within excited SMCs leads to the opening of voltage-gated Ca^2+^ channels, mainly via L-type Ca^2+^ channels [39,52], but also through T-type Ca^2+^ channels [53]. The finding that a transient elevation of cytosolic Ca^2+^ could be induced by K^+^ induced depolarization, combined with the fact that this phenomenon was inhibited in the presence of the Ca^2+^ channel blocker Cd^2+^, proved that myogenically differentiated MSCs and bladder SMCs, but not undifferentiated MSCs in which this effect was not seen, express functional voltage-gated Ca^2+^ channels which is essential for the activation of contraction in bladder smooth muscle [13].

The molecular identity of the observed functional Ca^2+^ currents in this study was confirmed. Similar to bladder SMCs, differentiated MSCs already at day 7 expressed ion channel genes encoding the Ca_v_1.2 L-type Ca^2+^ and Ca_v_3.2 T-type Ca^2+^ channels, as well as the large conductance Ca^2+^-activated K^+^ channels (BKCa). These findings are important since bladder SMCs depend on all of these ion channels types for contraction [38,39]. Like others [38], we show that BKCa channel was highly expressed in bladder SMCs, but also in differentiated bone marrow-derived MSCs. In line with these results both cell types responded by contracting in response to K^+^. The BKCa has been proposed to be the most important physiologically relevant K^+^ channel that regulates urinary bladder SMC function [38]. Like BKCa currents, T- and L-type Ca^2+^ currents have been found in SMCs from the human bladder [39,40,54], but also in other urological tissues including the proximal urethra [55] and prostate [56] where they regulate smooth muscle contraction. TGF-β1 may be responsible for the expression of these ion channels in differentiating MSCs since it increased the expression of these ion channels, as well as contractile SMC myogenic genes, in adipose-derived MSCs [28].

We not only show that both differentiated MSCs and SMCs reacted with a transiently elevated cytosolic Ca^2+^ response induced by K^+^ depolarization, but also confirmed that differentiated MSCs and bladder SMCs were significantly more contractile in response to KCl than undifferentiated MSCs. Instead of measuring the effects of cellular contraction on hydrogel dimensions, contraction of individual cells was measured. In order to accurately measure individual cellular dimensional changes within hydrogel constructs one would need to record and reconstruct z-stacks. This would severely limit the amount of cells that could be measured simultaneously. To achieve a sufficiently high *n* number we measured contraction of individual cells on top of hydrogels, allowing a relatively high number of cells situated within the same focus plane to be assessed, which bypasses the need for z-stack recording. Thus our approach allowed us to record changes in cellular dimensions and hence contraction on a large number of individually recorded cells. Significant changes in cellular length were within the range of -3.57 ± 0.52% for differentiated MSCs and -1.86 ± 0.37% for bladder SMCs. These percentages are similar to what has previously been shown for bladder SMCs [57], calculated as percentage in [58]. Based on all of these results we conclude that KCl treatment induced the contraction of differentiated MSCs, like bladder SMCs, and suggests that the contraction was due to the action of the contractile apparatus proteins and ion channels within the cells.

Collectively this study indicates that within 7–14 days of myogenic induction a large proportion (up to 60–80%) of the differentiating cells achieve maturity, express SMC markers and exhibit functional electrophysiological parameters, including the ability to contract. Contractile and synthetic SMCs, which represent the two ends of a spectrum of SMCs with intermediate phenotypes often present, clearly show different morphologies. This phenomenon, referred to as phenotypic modulation, is well characterized for primary vascular SMCs [42,59,60]. Hence vascular SMCs *in vitro* and *in vivo* can alternate from a functional, contractile phenotype to a proliferative ‘synthetic’ phenotype and often present as a continuum of these phenotypes [31,61]. Bladder SMCs have also been divided into phasic and tonic subtypes based on membrane properties and contractile behavior [13]. Hence, synthetic SMCs proliferate more than contractile SMCs, are dense, have a cobblestone morphology and contain many organelles essential to synthesize proteins [31,42]. In contrast, contractile SMCs are elongated, exhibit the shape of a spindle, express all of the contractile phenotypic markers that make up a functional contractile apparatus and respond to signals and stimuli that endorse cell contraction. SMCs with a contractile phenotype also predominantly express BKCa channels [62–65] and Ca_V_1.2 (L-type voltage-gated Ca^2+^) channels [66,67]. Therefore the differentiated MSCs derived in this work seem to mainly consist of the contractile phenotype, as they are spindle-shaped, express genes and proteins involved in SMC contraction including myogenic markers and functional ion channels, and contract when exposed to the K^+^ agonist.

This work constitutes a step forward in the pursuit of a better understanding of the expression of contractile myogenic markers in relation to the electrophysiological parameters over the course of *in vitro* differentiation of MSCs into smooth muscle-like cells and shows that electrophysiological characteristics of differentiated MSCs are comparable to bladder SMCs. This study could help in designing protocols aimed at urological regenerative medicine applications or where autologous smooth muscle cells are unavailable.

## References

[1] Atala A, Bauer SB, Soker S, Yoo JJ, Retik AB (2006) Tissue-engineered autologous bladders for patients needing cystoplasty. Lancet 367: 1241–1246. 16631879 10.1016/S0140-6736(06)68438-9

[2] Caione P, Boldrini R, Salerno A, Nappo SG (2012) Bladder augmentation using acellular collagen biomatrix: a pilot experience in exstrophic patients. Pediatr Surg Int 28: 421–428. 10.1007/s00383-012-3063-0 22350082

[3] Raghavan AM, Shenot PJ (2009) Bladder augmentation using an autologous neo-bladder construct. Kidney Int 76: 236 10.1038/ki.2009.81 19564862

[4] Bianco P, Cao X, Frenette PS, Mao JJ, Robey PG, Simmons PJ, et al (2013) The meaning, the sense and the significance: translating the science of mesenchymal stem cells into medicine. Nat Med 19: 35–42. 10.1038/nm.3028 23296015 PMC3998103

[5] Frenette PS, Pinho S, Lucas D, Scheiermann C (2013) Mesenchymal stem cell: keystone of the hematopoietic stem cell niche and a stepping-stone for regenerative medicine. Annu Rev Immunol 31: 285–316. 10.1146/annurev-immunol-032712-095919 23298209

[6] Lee CN, Jang JB, Kim JY, Koh C, Baek JY, Lee KJ (2010) Human cord blood stem cell therapy for treatment of stress urinary incontinence. J Korean Med Sci 25: 813–816. 10.3346/jkms.2010.25.6.813 20514298 PMC2877237

[7] Yamamoto T, Gotoh M, Kato M, Majima T, Toriyama K, Kamei Y, et al (2012) Periurethral injection of autologous adipose-derived regenerative cells for the treatment of male stress urinary incontinence: Report of three initial cases. Int J Urol 19: 652–659. 10.1111/j.1442-2042.2012.02999.x 22435469

[8] Gotoh M, Yamamoto T, Kato M, Majima T, Toriyama K, Kamei Y, et al (2014) Regenerative treatment of male stress urinary incontinence by periurethral injection of autologous adipose-derived regenerative cells: 1-year outcomes in 11 patients. Int J Urol 21: 294–300. 10.1111/iju.12266 24033774

[9] Klein G, Hart ML, Brinchmann JE, Rolauffs B, Stenzl A, Sievert KD, et al (2015) Mesenchymal stromal cells for sphincter regeneration. Adv Drug Deliv Rev 82-83C: 123–136. 10.1016/j.addr.2014.10.026 25451135

[10] Kim JH, Lee SR, Song YS, Lee HJ (2013) Stem cell therapy in bladder dysfunction: where are we? And where do we have to go? Biomed Res Int 2013: 930713 10.1155/2013/930713 24151627 PMC3787556

[11] Herrera-Imbroda B, Lara MF, Izeta A, Sievert KD, Hart ML (2015) Stress urinary incontinence animal models as a tool to study cell-based regenerative therapies targeting the urethral sphincter. Adv Drug Deliv Rev 82-83C: 106–116. 10.1016/j.addr.2014.10.018 25453264

[12] Hart ML, Izeta A, Herrera-Imbroda B, Amend B, Brinchmann JE (2015) Cell Therapy for Stress Urinary Incontinence. Tissue Eng Part B Rev 21: 365–376. 10.1089/ten.TEB.2014.0627 25789845

[13] Andersson KE, Arner A (2004) Urinary bladder contraction and relaxation: physiology and pathophysiology. Physiol Rev 84: 935–986. 15269341 10.1152/physrev.00038.2003

[14] Doughty DB (2005) Urinary and fecal incontinence: current management concepts: Mosby; 3 edition 656 p.

[15] Jack GS, Zhang R, Lee M, Xu Y, Wu BM, Rodríguez LV (2009) Urinary bladder smooth muscle engineered from adipose stem cells and a three dimensional synthetic composite. Biomaterials 30: 3259–3270. 10.1016/j.biomaterials.2009.02.035 19345408 PMC2744495

[16] Kanematsu A, Yamamoto S, Iwai-Kanai E, Kanatani I, Imamura M, Adam RM, et al (2005) Induction of smooth muscle cell-like phenotype in marrow-derived cells among regenerating urinary bladder smooth muscle cells. Am J Pathol 166: 565–573. 15681839 10.1016/S0002-9440(10)62278-X PMC1602323

[17] Tian H, Bharadwaj S, Liu Y, Ma H, Ma PX, Atala A, et al (2010) Myogenic differentiation of human bone marrow mesenchymal stem cells on a 3D nano fibrous scaffold for bladder tissue engineering. Biomaterials 31: 870–877. 10.1016/j.biomaterials.2009.10.001 19853294 PMC2787773

[18] Gong Z, Calkins G, Cheng EC, Krause D, Niklason LE (2009) Influence of culture medium on smooth muscle cell differentiation from human bone marrow-derived mesenchymal stem cells. Tissue Eng Part A 15: 319–330. 10.1089/ten.tea.2008.0161 19115826 PMC2716410

[19] Bajpai VK, Mistriotis P, Andreadis ST (2012) Clonal multipotency and effect of long-term in vitro expansion on differentiation potential of human hair follicle derived mesenchymal stem cells. Stem Cell Res 8: 74–84. 10.1016/j.scr.2011.07.003 22099022 PMC3222855

[20] Harris LJ, Abdollahi H, Zhang P, McIlhenny S, Tulenko TN, DiMuzio PJ (2011) Differentiation of adult stem cells into smooth muscle for vascular tissue engineering. J Surg Res 168: 306–314. 10.1016/j.jss.2009.08.001 19959190 PMC2888621

[21] Williams C, Xie AW, Emani S, Yamato M, Okano T, Emani SM, et al (2012) A comparison of human smooth muscle and mesenchymal stem cells as potential cell sources for tissue-engineered vascular patches. Tissue Eng Part A 18: 986–998. 10.1089/ten.TEA.2011.0172 22145703

[22] Narita Y, Yamawaki A, Kagami H, Ueda M, Ueda Y (2008) Effects of transforming growth factor-beta 1 and ascorbic acid on differentiation of human bone-marrow-derived mesenchymal stem cells into smooth muscle cell lineage. Cell Tissue Res 333: 449–459. 10.1007/s00441-008-0654-0 18607632

[23] Bharadwaj S, Liu G, Shi Y, Markert C, Andersson KE, Atala A, et al (2011) Characterization of urine-derived stem cells obtained from upper urinary tract for use in cell-based urological tissue engineering. Tissue Eng Part A 17: 2123–2132. 10.1089/ten.TEA.2010.0637 21513463 PMC9836685

[24] Kurpinski K, Lam H, Chu J, Wang A, Kim A, Tsay E, et al (2010) Transforming growth factor-beta and notch signaling mediate stem cell differentiation into smooth muscle cells. Stem Cells 28: 734–742. 10.1002/stem.319 20146266

[25] Seeger T, Hart M, Patarroyo M, Rolauffs B, Aicher WK, Klein G (2015) Mesenchymal Stromal Cells for Sphincter Regeneration: Role of Laminin Isoforms upon Myogenic Differentiation. PLoS One 10: e0137419 10.1371/journal.pone.0137419 26406476 PMC4583377

[26] Antoon R, Yeger H, Loai Y, Islam S, Farhat WA (2012) Impact of bladder-derived acellular matrix, growth factors, and extracellular matrix constituents on the survival and multipotency of marrow-derived mesenchymal stem cells. J Biomed Mater Res A 100: 72–83. 10.1002/jbm.a.33230 21972045

[27] Felka T, Schäfer R, De Zwart P, Aicher WK (2010) Animal serum-free expansion and differentiation of human mesenchymal stromal cells. Cytotherapy 12: 143–153. 10.3109/14653240903470647 20141338

[28] Park WS, Heo SC, Jeon ES, Hong da H, Son YK, Ko JH, et al (2013) Functional expression of smooth muscle-specific ion channels in TGF-beta(1)-treated human adipose-derived mesenchymal stem cells. Am J Physiol Cell Physiol 305: C377–391. 10.1152/ajpcell.00404.2012 23761629 PMC3891216

[29] Ulrich C, Rolauffs B, Abele H, Bonin M, Nieselt K, Hart ML, et al (2013) Low osteogenic differentiation potential of placenta-derived mesenchymal stromal cells correlates with low expression of the transcription factors Runx2 and Twist2. Stem Cells Develop 22: 2859–2872. 10.1089/scd.2012.0693 PMC3804084 23763516

[30] Hamill OP, Marty A, Neher E, Sakmann B, Sigworth FJ (1981) Improved patch-clamp techniques for high-resolution current recording from cells and cell-free membrane patches. Pflugers Arch 391: 85–100. 6270629 10.1007/BF00656997

[31] Beamish JA, He P, Kottke-Marchant K, Marchant RE (2010) Molecular regulation of contractile smooth muscle cell phenotype: implications for vascular tissue engineering. Tissue Eng Part B Rev 16: 467–491. 10.1089/ten.TEB.2009.0630 20334504 PMC2943591

[32] Bühring HJ, Battula VL, Treml S, Schewe B, Kanz L, Vogel W (2007) Novel markers for the prospective isolation of human MSC. Ann NY Acad Sci 1106: 262–271. 17395729 10.1196/annals.1392.000

[33] Bieback K (2013) Platelet lysate as replacement for fetal bovine serum in mesenchymal stromal cell cultures. Transfus Med Hemother 40: 326–335. 10.1159/000354061 24273486 PMC3822281

[34] Pilz GA, Ulrich C, Ruh M, Abele H, Schäfer R, Kluba T, et al (2011) Human term placenta-derived mesenchymal stromal cells are less prone to osteogenic differentiation than bone marrow-derived mesenchymal stromal cells. Stem Cells Dev 20: 635–646. 10.1089/scd.2010.0308 21047215

[35] Ulrich C, Abruzzese T, Maerz JK, Ruh M, Amend B, Benz K, et al (2015) Human Placenta-Derived CD146-Positive Mesenchymal Stromal Cells Display a Distinct Osteogenic Differentiation Potential. Stem Cells Dev 24: 1558–1569. 10.1089/scd.2014.0465 25743703

[36] Wang GK, Calderon J, Wang SY (2008) State- and use-dependent block of muscle Nav1.4 and neuronal Nav1.7 voltage-gated Na+ channel isoforms by ranolazine. Mol Pharmacol 73: 940–948. 18079277 10.1124/mol.107.041541 PMC2275669

[37] Schmalhofer WA, Calhoun J, Burrows R, Bailey T, Kohler MG, Weinglass AB, et al (2008) ProTx-II, a selective inhibitor of NaV1.7 sodium channels, blocks action potential propagation in nociceptors. Mol Pharmacol 74: 1476–1484. 10.1124/mol.108.047670 18728100

[38] Petkov GV (2014) Central role of the BK channel in urinary bladder smooth muscle physiology and pathophysiology. Am J Physiol Regul Integr Comp Physiol 307: R571–584. 10.1152/ajpregu.00142.2014 24990859 PMC4166757

[39] Wegener JW, Schulla V, Lee TS, Koller A, Feil S, Feil R, et al (2004) An essential role of Cav1.2 L-type calcium channel for urinary bladder function. FASEB J 18: 1159–1161. 15132976 10.1096/fj.04-1516fje

[40] Fry CH, Jabr RI (2014) T-type Ca2+ channels and the urinary and male genital tracts. Pflugers Arch 466: 781–789. 10.1007/s00424-014-1446-x 24463704

[41] Hart ML, Izeta A, Herrera-Imbroda B, Amend B, Brinchmann JE (2015) Cell Therapy for Stress Urinary Incontinence. Tissue Eng Part B Rev In press. 10.1089/ten.TEB.2014.0627 25789845

[42] Rensen SS, Doevendans PA, van Eys GJ (2007) Regulation and characteristics of vascular smooth muscle cell phenotypic diversity. Neth Heart J 15: 100–108. 17612668 10.1007/BF03085963 PMC1847757

[43] Herman IM (1993) Actin isoforms. Curr Opin Cell Biol 5: 48–55. 8448030 10.1016/s0955-0674(05)80007-9

[44] Assinder SJ, Stanton JA, Prasad PD (2009) Transgelin: an actin-binding protein and tumour suppressor. Int J Biochem Cell Biol 41: 482–486. 10.1016/j.biocel.2008.02.011 18378184

[45] Winder SJ, Walsh MP (1993) Calponin: thin filament-linked regulation of smooth muscle contraction. Cell Signal 5: 677–686. 8130072 10.1016/0898-6568(93)90029-l

[46] Babu GJ, Warshaw DM, Periasamy M (2000) Smooth muscle myosin heavy chain isoforms and their role in muscle physiology. Microsc Res Tech 50: 532–540. 10998642 10.1002/1097-0029(20000915)50:6<532::AID-JEMT10>3.0.CO;2-E

[47] Muraki K, Imaizumi Y, Watanabe M (1991) Sodium currents in smooth muscle cells freshly isolated from stomach fundus of the rat and ureter of the guinea-pig. J Physiol 442: 351–375. 1665861 10.1113/jphysiol.1991.sp018797 PMC1179893

[48] Saleh S, Yeung SY, Prestwich S, Pucovsky V, Greenwood I (2005) Electrophysiological and molecular identification of voltage-gated sodium channels in murine vascular myocytes. J Physiol 568: 155–169. 16020462 10.1113/jphysiol.2005.090951 PMC1474751

[49] Shinjoh M, Nakaki T, Otsuka Y, Sasakawa N, Kato R (1991) Vascular smooth muscle contraction induced by Na+ channel activators, veratridine and batrachotoxin. Eur J Pharmacol 205: 199–202. 1667387 10.1016/0014-2999(91)90820-g

[50] Cox RH, Zhou Z, Tulenko TN (1998) Voltage-gated sodium channels in human aortic smooth muscle cells. J Vasc Res 35: 310–317. 9789111 10.1159/000025600

[51] Ratz PH, Berg KM, Urban NH, Miner AS (2005) Regulation of smooth muscle calcium sensitivity: KCl as a calcium-sensitizing stimulus. Am J Physiol Cell Physiol 288: C769–783. 15761211 10.1152/ajpcell.00529.2004

[52] Kajioka S, Nakayama S, McMurray G, Abe K, Brading AF (2002) Ca(2+) channel properties in smooth muscle cells of the urinary bladder from pig and human. Eur J Pharmacol 443: 19–29. 12044787 10.1016/s0014-2999(02)01593-5

[53] Fry CH, Sui G, Wu C (2006) T-type Ca2+ channels in non-vascular smooth muscles. Cell Calcium 40: 231–239. 16797698 10.1016/j.ceca.2006.04.027

[54] Sui GP, Wu C, Fry CH (2003) A description of Ca2+ channels in human detrusor smooth muscle. BJU Int 92: 476–482. 12930444 10.1046/j.1464-410x.2003.04356.x

[55] Hollywood MA, Woolsey S, Walsh IK, Keane PF, McHale NG, Thornbury KD (2003) T- and L-type Ca2+ currents in freshly dispersed smooth muscle cells from the human proximal urethra. J Physiol 550: 753–764. 12807987 10.1113/jphysiol.2003.043695 PMC2343068

[56] Sui GP, Wu C, Fry CH (2004) Ca2+ currents in smooth muscle cells isolated from human prostate. Prostate 59: 275–281. 15042603 10.1002/pros.20007

[57] Herrera GM, Heppner TJ, Nelson MT (2001) Voltage dependence of the coupling of Ca(2+) sparks to BK(Ca) channels in urinary bladder smooth muscle. Am J Physiol Cell Physiol 280: C481–490. 11171567 10.1152/ajpcell.2001.280.3.C481

[58] Hill-Eubanks DC, Werner ME, Heppner TJ, Nelson MT (2011) Calcium signaling in smooth muscle. Cold Spring Harb Perspect Biol 3: a004549 10.1101/cshperspect.a004549 21709182 PMC3181028

[59] Owens GK, Kumar MS, Wamhoff BR (2004) Molecular regulation of vascular smooth muscle cell differentiation in development and disease. Physiol Rev 84: 767–801. 15269336 10.1152/physrev.00041.2003

[60] Campbell JH, Campbell GR (2012) Smooth muscle phenotypic modulation—a personal experience. Arterioscler Thromb Vasc Biol 32: 1784–1789. 10.1161/ATVBAHA.111.243212 22815344

[61] Sobue K, Hayashi K, Nishida W (1999) Expressional regulation of smooth muscle cell-specific genes in association with phenotypic modulation. Mol Cell Biochem 190: 105–118. 10098977

[62] Neylon CB, Lang RJ, Fu Y, Bobik A, Reinhart PH (1999) Molecular cloning and characterization of the intermediate-conductance Ca(2+)-activated K(+) channel in vascular smooth muscle: relationship between K(Ca) channel diversity and smooth muscle cell function. Circ Res 85: e33–43. 10532960 10.1161/01.res.85.9.e33

[63] Archer SL, Huang JM, Reeve HL, Hampl V, Tolarova S, Michelakis E, et al (1996) Differential distribution of electrophysiologically distinct myocytes in conduit and resistance arteries determines their response to nitric oxide and hypoxia. Circ Res 78: 431–442. 8593702 10.1161/01.res.78.3.431

[64] Platoshyn O, Remillard CV, Fantozzi I, Mandegar M, Sison TT, Zhang S, et al (2004) Diversity of voltage-dependent K+ channels in human pulmonary artery smooth muscle cells. Am J Physiol Lung Cell Mol Physiol 287: L226–238. 15047570 10.1152/ajplung.00438.2003

[65] Cheong A, Wood IC, Beech DJ (2006) Less REST, more vascular disease? Regulation of cell cycle and migration of vascular smooth muscle cells. Cell Cycle 5: 129–131. 16357530 10.4161/cc.5.2.2310

[66] House SJ, Potier M, Bisaillon J, Singer HA, Trebak M (2008) The non-excitable smooth muscle: calcium signaling and phenotypic switching during vascular disease. Pflugers Arch 456: 769–785. 10.1007/s00424-008-0491-8 18365243 PMC2531252

[67] Rodman DM, Reese K, Harral J, Fouty B, Wu S, West J, et al (2005) Low-voltage-activated (T-type) calcium channels control proliferation of human pulmonary artery myocytes. Circ Res 96: 864–872. 15774856 10.1161/01.RES.0000163066.07472.ff

